# Over-the-Air Firmware Updates for Constrained NB-IoT Devices

**DOI:** 10.3390/s22197572

**Published:** 2022-10-06

**Authors:** Farouk Mahfoudhi, Ashish Kumar Sultania, Jeroen Famaey

**Affiliations:** 1 Optical Networks, Nokia Networks, 2018 Antwerp, Belgium; 2 Department of Computer Science, University of Antwerp and Imec, 2000 Antwerp, Belgium

**Keywords:** firmware update, OTA, NB-IoT

## Abstract

The Internet of Things (IoT) is being deployed to provide smart solutions for buildings, logistics, hospitals, and many more. It is growing with billions of connected devices. However, with such tremendous growth, maintenance and support are the hidden burdens. The devices deployed for IoT generally have a light microcontroller, low-power, low memory, and lightweight software. The software, which includes firmware and applications, can be managed remotely via a wireless connection. This improves flexibility, installation time, accessibility, effectiveness, and cost. The firmware can be updated constantly to remove known bugs and improve the functionality of the device. This work presents an approach to update firmware over-the-air (OTA) for constrained IoT devices. We used Narrowband IoT (NB-IoT) as the wireless communication standard to communicate between the managing server and devices. NB-IoT is one of the most promising low power wide area (LPWA) network protocols that supports more than 50k devices within a cell using a licensed spectrum. This work is a proof of concept demonstrating the usage of NB-IoT to update firmware for constrained devices. We also calculated the overall power consumption and latency for different sizes of the firmware.

## 1. Introduction

Billions of IoT devices are expected to work for more than ten years operating at very low-energy consumption considering their limited capabilities such as battery life, processing power, and memory. It means the microcontroller, sensors, and communication need to be low-power. These devices are controlled and monitored remotely using network connections, usually over the Internet. However, to connect these devices to the network, traditional power-hungry Wi-Fi or mobile technology cannot be used. We can use a low-power wide-area network (LPWAN) technology such as NB-IoT, which is a communication standard that provides low energy consumption, reliable connection and deep-indoor coverage for the device. There are two techniques, namely extended discontinuous reception (e-DRX) and power saving mode (PSM), which are applied in NB-IoT to lower the energy consumption of the device. In [1], we already determined that an NB-IoT device with a 5 Wh battery can operate for more than 12 years when transmitting at most one packet per day. The maintenance of these devices can be performed by updating the firmware periodically to upgrade features, resolve known security vulnerabilities, update supported protocols and fix existing software bugs. We focus in particular on firmware update mechanisms that can work on constrained NB-IoT enabled devices.

The firmware is usually stored in electrically erasable programmable read-only memory (EEPROM) or flash memory. The firmware of IoT devices has features such as working with real-time behaviour, deterministic execution, fast boot time, closed system with fixed-function, and fixed boot target [2]. The required activities for the firmware update can be categorized as management, availability, notification, transfer, and security. The update mechanism should not affect the user’s ordinary data communication. Depending on the devices, updating the firmware can be done manually with a Joint Test Action Group (JTAG) connected to the device, via an On-The-Go (OTG) cable, or via a serial port. However, over-the-air (OTA) updating using a wireless connection provides flexibility, remote accessibility, easy maintenance, and cost-effectiveness. The update process can be divided into four steps, (a) firmware generation, (b) distribution, (c) verification of its integrity and authenticity, and (d) installation on the device [3]. We adopt a client-server model where the server alone is responsible for sending updated firmware images, and also receiving device data. The update notification can be pushed by the server or can be pulled by the device (client) by periodically polling the server to check for any updates. This push or pull can be done manually or automatically. The manual update is time-consuming and error-prone because of the involvement of the user(s). Therefore, it is generally recommended to do it in a fully automated manner when updating many devices. However, to automatically receive or poll the update, the device can perform it at the end of the Tracking Area Updating (TAU) period or needs to wake up outside its sleeping mode (PSM), which impacts its battery life. Based on our evaluation, we can say that the proposed system works well for various use cases in the domains of smart cities and building automation.

The major contribution of our work is the development of a remote firmware update architecture using NB-IoT as a low-power energy consumption network and effectively transferring the firmware images to a large number of devices by having a negligible impact on the battery lifetime. The remainder of the paper is organized as follows. In Section 2, we provide an overview of the related literature. Section 3 describes an overview of our system architecture and the prototype. Section 4 presents the observation from the implemented prototype, and we conclude our discussion in Section 5.

## 2. Related Work

The IoT device firmware update generally consists of the development of a management framework, device design for supporting the remote update, and transferring the firmware remotely. A study of the firmware update for embedded IoT systems is described by Nikolov [4]. He showed a way of updating the management software of an embedded system remotely from the cloud. Before transferring the updated image, the devices need to be notified of its transfer. Another system design has been proposed by Chandra et al. [5] for a lightweight mesh-based network protocol to update an interconnected device firmware that requires low power consumption.

Smart systems use technologies such as Wi-Fi, Bluetooth, and ZigBee for software update transfer. Texas Instruments has developed the Z-Stack protocol stack-based wireless firmware updating technique, which can meet the basic need of ZigBee nodes [6]. A few researchers have attempted to optimize this standard technique. Shen et al. propose one such scheme based on tree networks to solve efficiency problems in the process of broadcasting upgrades [7]. In 2017, Feng [8] proposed a Distributed Priority Page-request algorithm for updating via ZigBee-based networks.

However, other communication standards for low-powered devices include LoRaWAN and NB-IoT. LoRaWAN restricts the maximum amount of data communication per time period, making the system slower for updating. The updates interfere with data communication and the device becomes non-communicable for a long time. On the other hand, the NB-IoT standard supports group services with the multimedia broadcast multicast service (MBMS), which can help send update notifications. The single cell-point to multi-point (SC-PTM) framework is one of the approaches that extend MBMS to provide group communications within a single cell [9]. More recently, it was also standardized in NB-IoT [10].

A few works analyzed the NB-IoT firmware update theoretically. Feltrin et al. [11] compared the performance achieved with unicast DL and SC-PTM transmission schemes. They concluded that the data latency in an NB-IoT network is affected by the number of UEs in the unicast case, but not with SC-PTM. Gonzalez [12] analyzes the transmission time for large firmware size of 1 MB for different coverage conditions of the NB-IoT network. However, this work does not consider the power consumption and focuses only on the theoretical analysis. Recently, Nikic et al. [13] presented a working firmware update solution using custom-designed edge nodes, focusing on the update timings for non-encrypted and encrypted protocols, whereas our work focuses on a prototype design for firmware update and analyzes not only the transmission latency but also the battery power consumption on a live NB-IoT network in Belgium.

Some works have focused on calculating the power consumption of NB-IoT modules from different vendors on different deployed networks. Alobaidy et al. [14] performed the experiment using Pycom FiPy as NB-IoT UE and measured its power consumption on MAXIS, which is a mobile network operator in Malaysia. Khan et al. [15] evaluated the power consumption of two different evaluation boards of Quectel BG96 LPWAN module from Avnet Silica NB-IoT sensor shield and Quectel UMTS & LTE EVB Kit. They performed their experiments in two different NB-IoT operators in Estonia. It is observed that the power consumption varies for different operators, whereas, the Avnet board consumes less power in all the radio states. Yeoh et al. [16] worked with NB-IoT module from Quectel and u-blox but on virtual EPC from Huawei. It is observed that the network attach time is much lower on the virtual EPC compared to the experiments on a deployed live network. Michelinakis et al. [17] also used Quectel and u-blox modules and observed that the u-blox module consumes more power compared to Quectel. The results from the experiments from [18] also show that the UE (u-blox module) in a private network consumes less power than the commercially deployed one. However, these works focus on evaluating the power consumption for fixed data rate and mostly uplink data without considering the firmware update process.

There are many platforms such as Mender [19], ARM Pelion [20], Balena [21], Particle [22], and AWS IoT-FreeRTOS [23] that offer firmware update functionality for IoT devices. These platforms focus on providing reliable, secure and robust solutions with features such as update failure management, rollback, reduced downtime, secure communication, authenticity and integrity. All of these platforms mainly use Wi-Fi and cellular networks. Considering the power consumption of these technologies, they are not suitable for low-power IoT devices deployed with a non-chargeable battery. Therefore, the platforms use a powered-gateway device in-middle and low-power short-range communication technology such as BLE and IEEE 802.15.4 to save power at the end devices. Although we use similar characteristics to these platforms to provide a reliable, secure and robust solution, we instead exclusively consider low-power NB-IoT as a long-range communication technology. Therefore, our solution can be useful for devices with non-chargeable batteries deployed in hard-to-reach areas, where the deployment of an additional short-range mains-powered gateway might not be feasible. ARM Pelion also provides the NB-IoT communication support by enabling differential updates, but they did not evaluate the impact on the device battery life on performing the firmware updates. Although the concept of conditional updates is implemented by ARM Pelion which enables the device to accept the updates based on pre-defined conditions, such as at minimum battery level, we evaluate the power consumption of the system for different firmware sizes.

There are a few solutions available online [24,25,26]. The first one [24] uses 6LoWPAN to transfer the firmware but also needs a gateway to download it from the server. The device memory is partitioned into two sections. The executing image resides in the first section, and the second section is used to temporarily store the image being downloaded. However, if an issue occurs during the firmware download such as missing segments or bad checksum, there is no way for the failure recovery. Other solutions [25,26] consider LoRa as a firmware transfer medium and provide a solution without having any dedicated local gateway. However, with the limitations on the data rate and the maximum number of messages, it can take up to 9 hours to transfer 100 kB firmware. Therefore, we aim to avoid these bottlenecks of installing a gateway or having limitations on the number of messages to be transmitted by proposing a reliable solution using public NB-IoT networks.

## 3. System Architecture

### 3.1. System Overview

**Figure 1:**
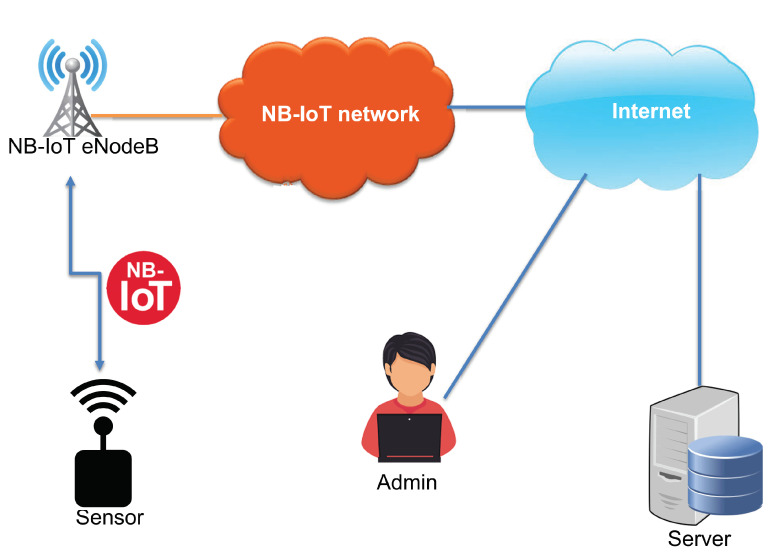
Network architecture.

In this section, the system architecture is described. We define the system with end-nodes, and the NB-IoT network is used as a communication interface between them, as shown in Figure 1. The following end-nodes are considered:

- **Sensor**: It is a device connected to the NB-IoT network and senses various data such as temperature, humidity, and GPS position. It sends the sensed data periodically to a server via the NB-IoT network. The sensor needs to update its functionalities and becomes a target for the wireless firmware update over NB-IoT.
- **Server**: It is a self-deployed server connected to the Internet or a cloud service. It is used to maintain the database of the system and store the sensors’ identities and measurements. In addition to that, the server manages firmware versions and handles the sensors’ firmware update process. The update process can be triggered automatically by the server application or manually by the administrator using a web interface. Before transferring the firmware update file, the server notifies all the registered sensors about the upcoming files via NB-IoT.

The update image can be a partial update, differential binary patching, or full firmware image replacement at once. There could be two modes of firmware update, as below:

- **Push firmware update**: Whenever a new firmware version is available, the server starts the update process.
- **Pull firmware update**: In this case, the sensor or the user at the sensor-side can initiate the update process. Practically, the sensors have a dedicated button that needs to be long pressed at bootup to start the firmware update.

The firmware update operations should fulfill the following requirements.

- *Notification about the update*: The availability of a new firmware version should be notified to all the eligible sensors, which is done by the server.
- *Version control at server-side*: The server should manage all the firmware versions so that the firmware rollback is possible.
- *Authenticity of update file source*: The sensors should accept the firmware updates only from authenticated servers.
- *Reliability in transfer*: Packet loss or file corruption can affect firmware update operations and can fail the process. Therefore, the file transfer protocol should be reliable, which is generally controlled by the destination-feedback mechanism.
- *Integrity of the firmware-image*: The complete transfer of the firmware should be verified in a way so that a corrupted file can be rejected.
- *Backward compatibility*: The sensors should be able to revert the firmware version to the last running one. To support backward compatibility, the sensor needs to have a large flash memory, which could affect the cost of its deployment.
- *Hindrance to the user data communication*: The firmware update files should be transferred so that they do not block the main data communication processes.
- *Automated process*: The complete process of updating should be automated such as the sensors should be able to boot automatically to the new firmware; it should check the integrity, negotiate the timing of the update, and much more.
- *Secure Channel*: The communication between the server and the sensor should be secure. A secure handshake should be performed to negotiate security parameters, and transmitted data should be encrypted. Furthermore, the firmware can be signed for additional security.

As mentioned before, the sensor device must manage its main function, which is reporting various sensors’ data to the server via the NB-IoT network while the firmware update process is running. There are two approaches to performing it. The first approach is referred to as **background firmware update**. The software running at the sensor handles both the data reporting and the firmware update in parallel. The background update approach is favourable for large firmware updates. The second approach is a **foreground firmware update**, in which two different software agents or threads are separately responsible for the data reporting and the firmware update. In our architecture, we used the second approach: the two agents are the application and the bootloader. The application is responsible for collecting data and reporting them to the server via the NB-IoT network, whereas the bootloader is a program that handles the firmware update operations. This way we separate responsibilities between the two agents which results in easy maintenance of the code and less code change in the application.

**Figure 2:**
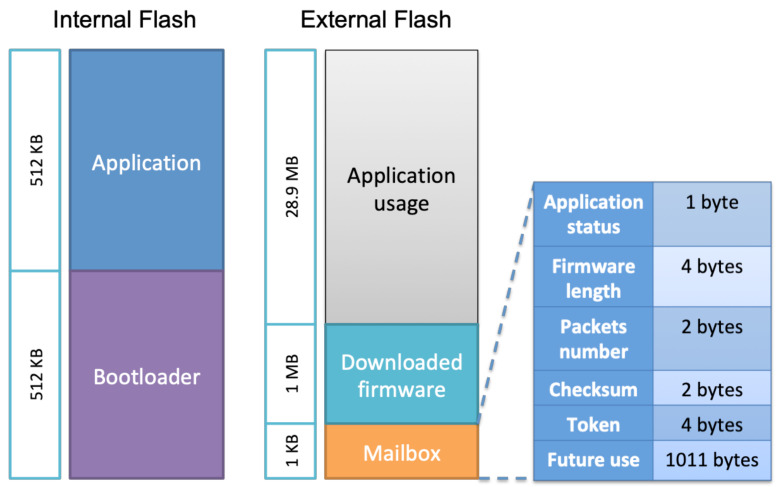
Internal and external flash memory layout.

Both of them are stored in the sensor flash memory. The flash memory layout is shown in Figure 2. We defined a memory range named *Mailbox*, which enables data sharing between these two agents. When the sensor application receives a firmware update notification from the server, it stores the notification parameters in the mailbox and jumps to the bootloader. Whenever the bootloader finds a firmware update request in the mailbox, it starts the firmware update process. The complete procedure is explained in the next sections. Our focus in this article is on the firmware update process rather than on data communication.

### 3.2. System Prototype

**Figure 3:**
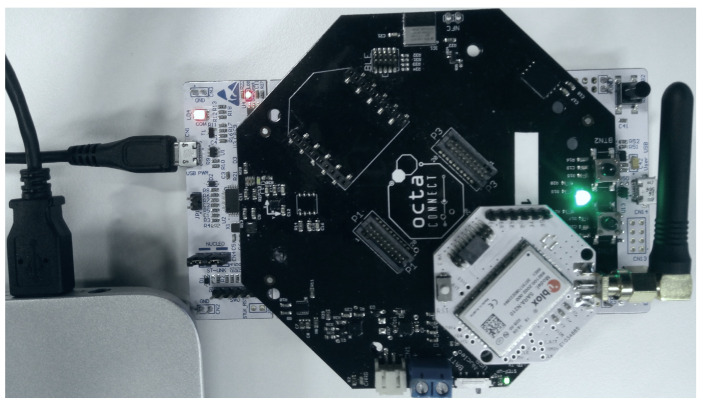
Device Setup prototype.

**Figure 4:**
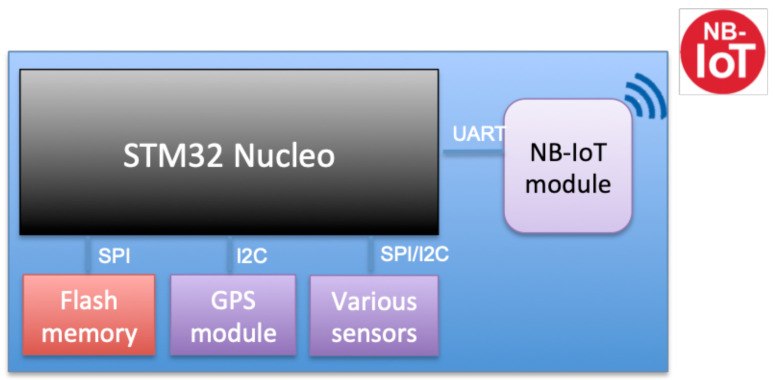
Sensor device components.

In this section, we describe the prototype designed to implement the required functionalities. Our aim is to show a proof of concept that NB-IoT can be used for the firmware update of IoT devices. Therefore, a prototype is presented without considering optimization and encryption. Our solution is modular and open to including additional features. Figure 3 and Figure 4 show the sensor components, which are described below:

- **STM32 Nucleo**: The development board STM32 Nucleo L496ZG is used to build the prototype. It runs both the sensor application and the bootloader for data reporting and firmware updates. It has many peripherals, and internal flash memory of one megabyte (MB) that can host both the application and the bootloader firmware [27].
- **NB-IoT module**: It is the communication module used to receive the firmware update file from the server using the NB-IoT network. It can also be used to exchange user data between the device and the server. We have used a SARA-N210 [28] based module as an NB-IoT device that is connected to the STM32 Nucleo board via UART and controlled via AT commands. The operator issues SIM cards for the sensors to access the network. Each SIM card has a unique International Mobile Equipment Identity (IMEI) that is used to identify the devices.
- **Octa Extension board**: A custom board plugged in the STM32 Nucleo GPIO socket that provides different connectors where various sensors and modules can be added.
- **Flash memory**: This is an extended external non-volatile memory and is used to store sensor application data and the downloaded firmware segments when performing the firmware update. It also hosts the mailbox to share data between the application and the bootloader. We have used S25FL256 flash memory [29] of 32 MB. It is soldered to the Octa Extension board and controlled via SPI from the STM32 Nucleo board.
- **Sensor modules**: There can be many sensor modules plugged into the Octa extension board and controlled by the STM32 Nucleo board using SPI, I2C, or UART. These modules are used to gather data such as GPS coordinates, temperature, and humidity.

### 3.3. Flash Memory Layout

This section describes the storage layout of the bootloader and application entities, and the management of the flash memories to perform the firmware update operations. The memory layout can be visualized as shown in Figure 2. Since the STM32 Nucleo L496ZG flash size is 1 MB, we have split it into two equal sections (512 KB) to use it for the sensor application firmware and to load the bootloader. The locations of the entities are defined in their linker script. The starting addresses of the bootloader and the sensor are provided to both the programs, i.e., bootloader and application, so the CPU can jump from bootloader to application or vice versa. It is required to have at least the bootloader running on the sensor, which can download the application if it is not flashed. Upon booting up the sensor, the bootloader detects the non-existence of the application and can initiate the firmware update process.

We chose to use the external flash to store firmware update-related data, in addition to other usage needed by the application. The external flash is divided into three sections as follows:

- **Application usage**: This section is dedicated to the sensor usage such as storing data or future usage to implement new additional features.
- **Downloaded firmware**: This area is used by the bootloader to store the downloaded firmware from the server during the firmware update process and before moving it to the internal flash. The firmware is sent from the server in segments, so the bootloader stores each one in this area using a buffering mechanism allowing it to be stored in contiguous blocks.
- **Mailbox**: As already explained, it is used to share data between the application and the bootloader regarding firmware update requests and application status. When a firmware update notification is received from the server, the sensor application updates this area that contains various fields such as *application status* which represents the current state of the application as defined in Table 1, *firmware length*, *packet number* to know the next expected segment counter, *firmware checksum* to validate its integrity, and *token* which is used to establish a firmware update session between the bootloader and the server. A token is generated by the server dedicated to a sensor and sent with the firmware update notification. Furthermore, some memory areas are reserved for future use.

### 3.4. Firmware Update Procedure and Application Data

**Figure 5:**
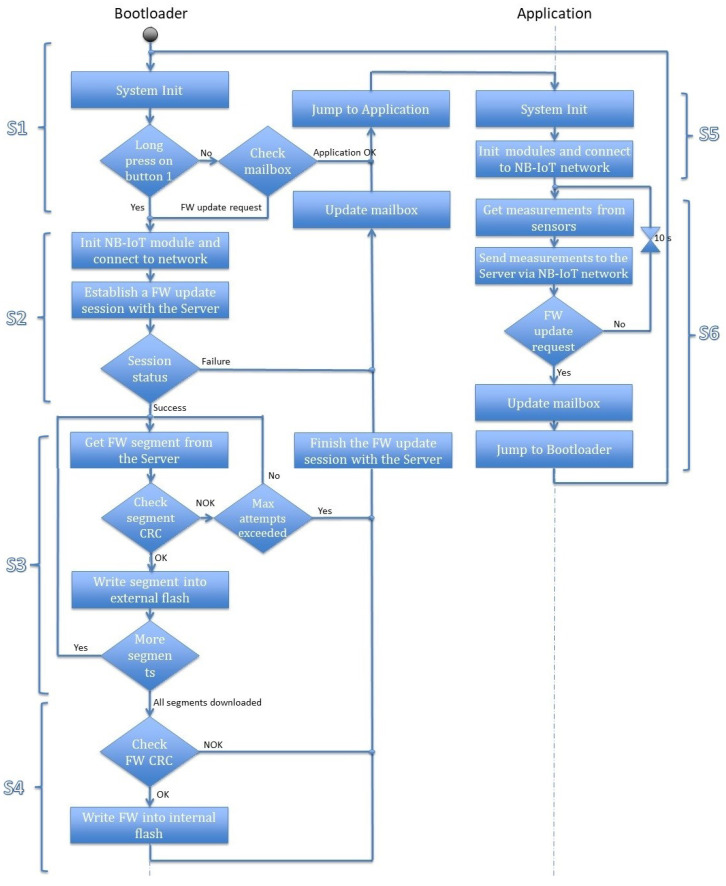
Firmware update concept.

This section discusses the firmware update procedure in detail. The activity diagram is presented in Figure 5, which describes the concept of the firmware update mechanism. We have divided the overall procedure into six steps, as explained below in which the first four steps (S1–S4) represent firmware updates and the last two steps (S5, S6) focus on application data transfer.

**(S1)** The CPU starts the execution from the bootloader address at the start of the boot up process. It begins by initializing the system and the required modules. It loads the mailbox data from the external flash and checks the application status field. If the status is *OK*, the CPU jumps to the application address and performs the actions from step *S5*. However, if the status is *Firmware update request* or a long press on the update button is detected, the bootloader executes the firmware update operation and starts executing step *S2*. On the other hand, if the application status field is *Firmware Downloaded*, which means that the firmware file is downloaded successfully from the server and written to the external flash but not yet written into the internal flash, the bootloader executes the instructions starting from step *S4*.

**(S2)** The bootloader initializes the NB-IoT module and connects to the NB-IoT network. It accesses the token that is sent from the server in the firmware update notification. This token is stored in the mailbox associated with the IMEI of the sensor. In the current session, the device uses this token to establish a firmware update session from the server. In the case of *pull firmware update* mode, the sensor uses the saved token from its last firmware update session. The server checks the identity of the sensor and uses it to validate the session request. However, for a new sensor device that would not have any token, it can initiate the request using its IMEI number. Upon receiving the request, the server checks its database to verify if any established previous bootstrap firmware update session exists from the requested IMEI number. It generates a new token and confirms the firmware update request. Then, the bootloader starts downloading the firmware from the server by following the instructions in step *S3*. Otherwise, it sets the application status to *Error(x)* to reset the *Firmware update request*. If the failure occurs while establishing the NB-IoT connection x is 1, if it fails during receiving the new token x is set to 2, and to 3 if the server is unable to verify the token. The server updates its database with the firmware update operation status, to track each sensor.

**(S3)** The bootloader retrieves the number of firmware segments from the mailbox that were already received from the server before it starts the reception process. The device can check its energy level and request the server to send the firmware segments one by one. The server responds each time with the requested segment file and its checksum. The bootloader verifies if the checksum received matches with the calculated checksum. If the checksum matches, the received segment is written to the *Downloaded Firmware* slot of the external flash. This operation uses buffering to guarantee that segments are written in contiguous blocks of the flash memory. If all the segments are downloaded, the bootloader executes the instructions in step *S4*. If the checksum does not match, the bootloader re-requests the same segment from the server. The mismatching of the checksum indicates that the downloaded segment is corrupted or downloaded incompletely. The device also maintains a number of maximum retry attempts to request the same segment to avoid infinite requests from the bootloader. After reaching the maximum number of attempts, the bootloader closes the firmware update session with the server. It sets the application status to *Error(4)* to reset the firmware update request. The server updates its database with the failure reason.

**(S4)** This step starts when all the firmware segments are downloaded, i.e., the complete firmware image is received. The successful completeness is verified by comparing the checksum for the complete firmware file with the received value from the server. The bootloader writes the downloaded firmware to the internal flash memory in the application section on successful verification. It also closes the firmware update session with success status, sets the application status field back to *OK*. Otherwise, if the checksum does not match, the bootloader closes the firmware update session with the server with the wrong firmware reason. It sets the application status to *Error(5)* to reset the firmware update request. Enhancement concerning the processing of this case is left for future work. The server updates its database with the firmware update operation status to know which sensors successfully updated their firmware, and which ones failed.

**(S5)** The application initializes the system, the sensors and the NB-IoT module, and connects to the NB-IoT network. Then it sends an identification message to the server containing the IMEI of the sensor, IP address of the NB-IoT module, Application software version, and timestamp. After that, step *S6* is executed.

**(S6)** The application starts gathering the sensors’ values periodically at the default period and sends them to the server, which stores them in its database. When a firmware update is triggered, the server sends a firmware update notification to the sensor containing the size of the firmware, number of packets, checksum, and a token. In this case, the application writes this information in the mailbox and sets the application status field to *Firmware update,* then jumps to the bootloader to restart step *S1*.

**Figure 6:**
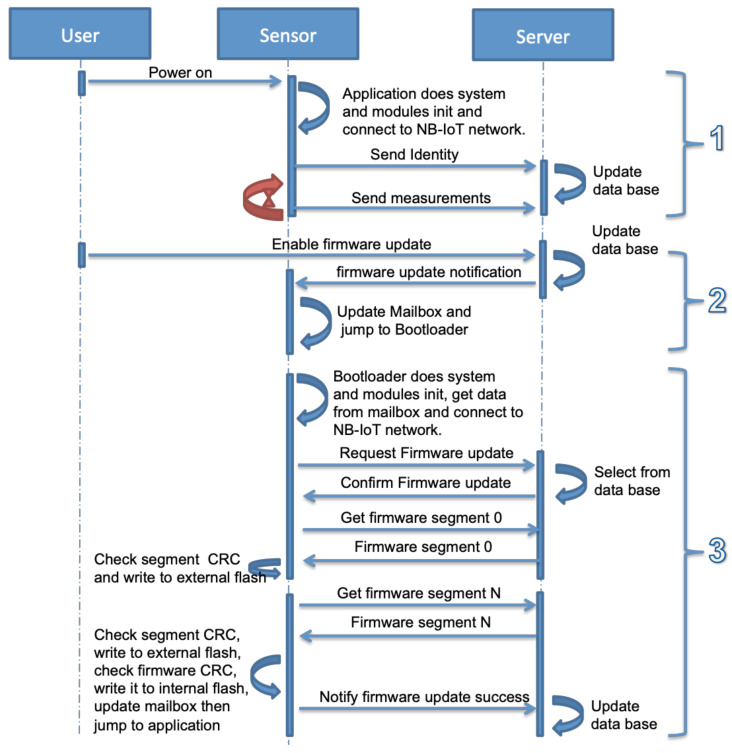
Firmware update mechanism.

The exchanged messages between the sensor and the server in normal operations and during the firmware update are shown in Figure 6. This process can be divided into three steps as described below.

**Figure 7:**
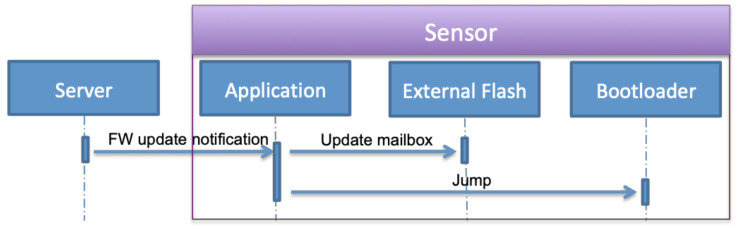
Firmware update notification operations.

**Figure 8:**
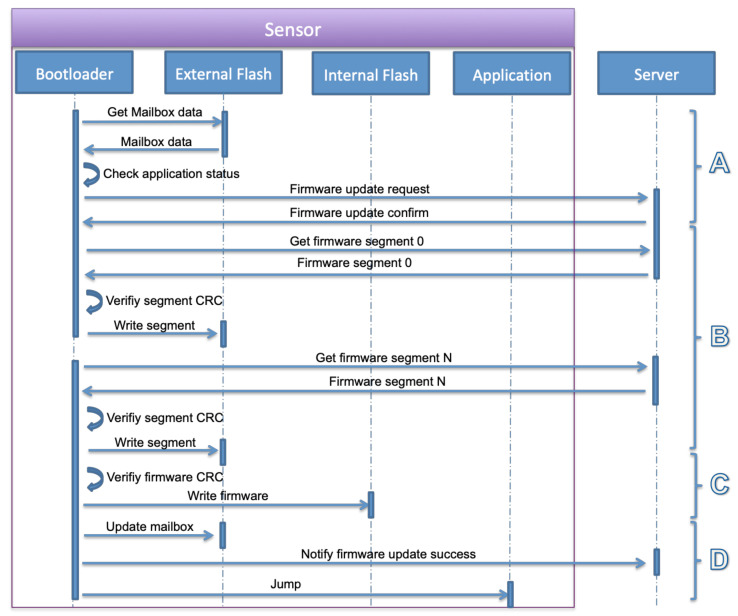
Bootloader firmware update operations.

- The user powers on the sensor. Its bootloader starts the application after checking the OK status of the application in the mailbox. The application initializes the system, the sensors and the NB-IoT module, and connects to the NB-IoT network. It sends an identification message to the server containing the IMEI of the sensor, the IP address of the NB-IoT module, the application software version, and the timestamp. Later, it starts recording the sensors’ data and sends it periodically to the server.
- The second step is enabling firmware updates. When it is enabled by the user, the server sends a firmware update notification to the sensor containing the size of the firmware, number of packets, checksum, and a token. As shown in Figure 7, the application writes this information in the mailbox and sets the application status field to the firmware update, then jumps to the bootloader.
- The final step is the bootloader operations. The bootloader initializes the system and required modules, loads the mailbox data from the external flash and checks the application status field. The status is firmware update request, the bootloader executes the firmware update operation as described in S3 and S4. These steps are detailed in Figure 8 that include firmware segments download, writing segments in external flash memory, writing firmware in internal flash memory, and finally notifying the successful update operation and jumping to a new application.

### 3.5. Server-Sensor Messages Format

The communication between the sensor and server is established using UDP sockets. The server can be configured to listen on a particular UDP port. We define some specific command formats for each message exchanged over the NB-IoT channel. They are presented in Table 2. The firmware is segmented into the maximum packet size of 1280 bytes so that to avoid IP fragmentation.

### 3.6. Software Architecture

In this section, we describe the software stacks of each entity of the system. As mentioned before, these consist of the sensor running the application and the bootloader, and the server.

#### 3.6.1. Sensor

**Figure 9:**
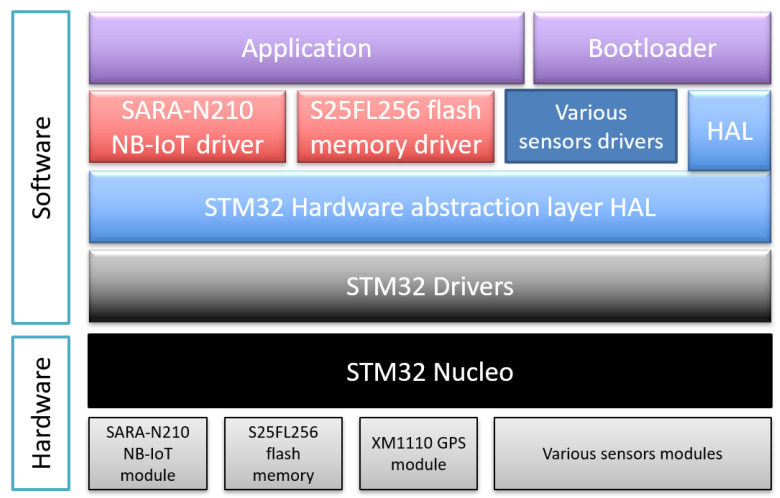
Application and bootloader software architecture.

The software architecture of the sensor application and bootloader running on the STM32 Nucleo is described in Figure 9. It consists of C/C++ files using different object-oriented classes and is divided into different layers. The following are the key software components involved in making the sensor and its firmware update functional.

- *Application:* The main program of the sensor application. It initializes the system, the sensors and the NB-IoT module, and connects to the NB-IoT network. Then it starts gathering data from the sensors and sends them periodically to the server via the NB-IoT network.
- *Bootloader:* The main program of the sensor bootloader. It initializes the system and reads the application status in the mailbox. If the status is OK, it jumps to the application. If not, it initializes the NB-IoT module and connects to the NB-IoT network. Then it starts performing the firmware update operations.
- *HAL:* STM32 standard hardware abstraction layer assuring the portability of the software applications between different STM32 boards.
- *SARA-N210 NB-IoT driver:* A C++ driver class that configures and manages the communication over NB-IoT of the SARA-N210 NB-IoT module via AT commands using the serial port. It uses the standard STM32 serial driver.
- *S25FL256 flash memory driver:* A C++ driver class that initializes the S25FL256 external flash memory and manages the read and write operations via SPI. It uses the standard STM32 SPI driver.
- *Various sensors drivers:* The temperature, GPS, humidity and other sensors drivers are used to initialize the sensors and read their values.
- *STM32 drivers:* Standard STM32 peripherals drivers such as I2C, SPI, UART, etc...

#### 3.6.2. Server

**Figure 10:**
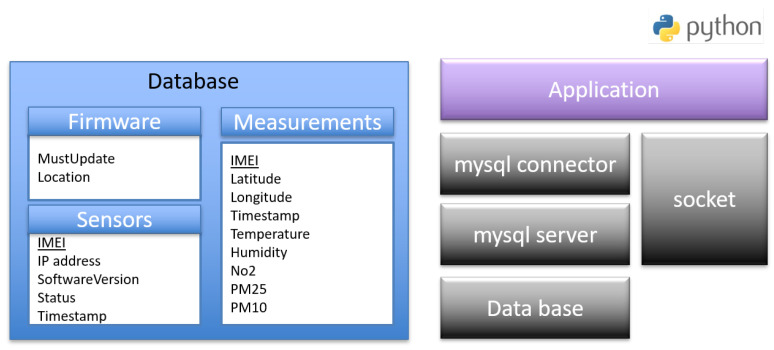
Server software architecture.

The software architecture of the server that is hosted on a dedicated machine or cloud machine publicly accessible via the Internet is described in Figure 10. The configuration and libraries required to configure the server are mentioned below.

- *socket:* The standard Python low-level networking interface library.
- *mysql connector:* It is used as a standardized database driver for MySQL database.
- *mysql server:* A database server that hosts the databases and manages all the clients’ requests.
- *database:* It stores sensors’ information in three tables. The first one is *Sensors* which stores the IMEI, the IP address, the software version, the last firmware update status and the timestamps of a sensor. The second one is *Measurement* table which stores IMEI, power, timestamps of the measurements and the values of the different sensors. The third one is *Firmware* table which contains the path of the recent firmware and an enable field of the download.
- *Application:* The script that stores all the sensor’s measurements in the database and handles firmware update operations of the sensors. It listens on UDP sockets and parses received messages from the sensors. It puts sensors’ identities and measurements, respectively, in the sensors and measurements database tables. It also triggers the firmware update of the sensors and handles the exchange with the sensors’ bootloaders during the process. It runs as a daemon to be active on booting up the server.

## 4. Experimental Validation

### 4.1. Evaluation Criteria

Experiments were conducted for different sizes of firmware, ranging from 2 to 1000 KB which is sufficient for many IoT devices [30,31]. We use the NB-IoT network of Orange in Belgium to evaluate our architecture design, and measure power consumption and transmission latency. As mentioned in Section 3.4, the firmware update steps are divided into four parts, (a) network connection setup and getting a firmware update notification, (b) requesting a firmware update, (c) receiving firmware segments, and (d) firmware installation and update notification.

#### 4.1.1. Power Consumption

**Figure 11:**
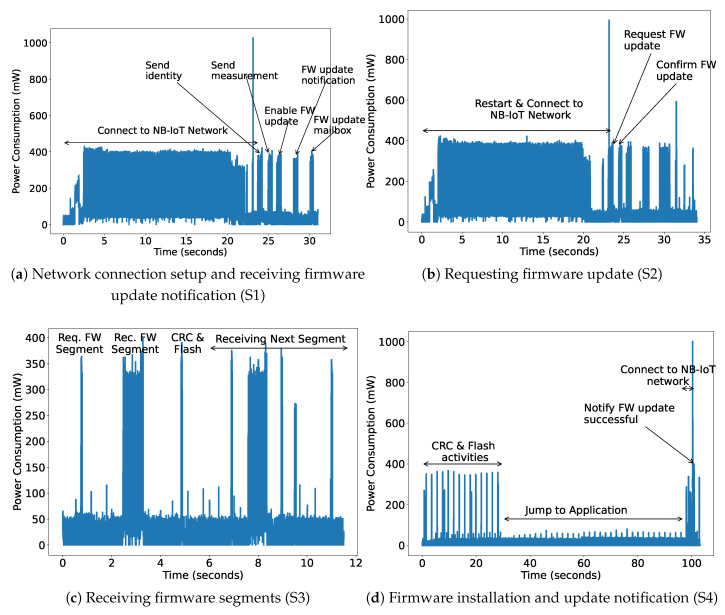
Power consumption of Firmware Update process.

The power consumption of the complete setup is measured using the off-the-shelf Nordic-Power Profiler Kit-II (PPK-II), which has a resolution of 0.1 μA [32]. We install the application and the bootloader using USB and later power it using PPK-II with 5V, the same as the battery power supply. The power consumption is measured for all the steps as shown in Figure 11. As shown in Figure 11a, the first step for the NB-IoT device is to turn on and connect to the network, which consumes the maximum time and power. Next, it sends its identity and first sensor data. The server can decide to send the notification to update the firmware by checking the current received version. The NB-IoT device updates its mailbox accordingly. All these steps take around 31.01 s and consume 217.44 mW power. After this step, the device jumps to the bootloader and therefore needs to re-establish the network connection as shown in Figure 11b. The device receives a different IP address on each reboot. However, now checking its mailbox, it will request the server to send the firmware update. The firmware is sent in segments according to the configured maximum packet size (1280 bytes). Then, the server confirms it, mentioning the number of segments. Both these steps take a long time due to the network connectivity task and therefore also consume a large amount of power, as shown in Table 3. Now, the next step is to receive all the segments, check the CRC, and save them in external flash. This activity for one FW segment consumes 44.85 mW. The last step is to check the downloaded firmware CRC, flash it into internal memory, update the mailbox, jump to the application and start sending the sensor data. It takes more than a minute to safely switch to the application. However, at this time the NB-IoT radio is in an e-DRX or Idle state. In this state, it periodically does paging and consumes an average of 9.62 mW power.

**Figure 12:**
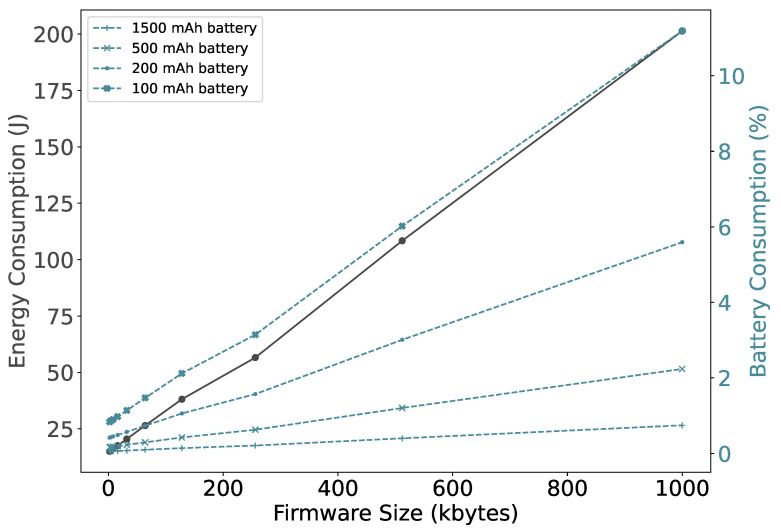
Bootloader firmware update operations.

Now, consolidating all the steps, we measure the device energy consumption as shown in Figure 12. A smaller firmware size results in fewer segments that need to be transferred and therefore consumes less energy. The energy consumption increases thirteenfold when the size of the firmware increases from 2 to 1000 KB. However, it does not vary that much, for the size up to 16 KB, which is also reflected in the battery consumption. As expected, if the battery is small, firmware updates have a relatively large impact. Specifically, a firmware update of 1000 KB, consumes around 11% of the total energy stored in a 100mAh battery. However, NB-IoT devices are typically powered by batteries with a capacity of 1500 mAh or more [33,34]. It can be observed that using these battery sizes the impact of performing a firmware update is less than 0.75%.

It is observed that NB-IoT consumes around 38 J to transfer 120 kB firmware, while the LoRa network consumption varies between 3 J and 250 J depending on the considered data rate [35]. Moreover, LoRa network firmware update transmissions observe packet losses up to 10%. The NB-IoT experiments are performed by placing the device in a room with good signal strength where the RSSI value is between −85 and −80 dBm. No packet losses are observed.

#### 4.1.2. Transmission Latency

The total firmware transmission latency includes the latency of all the states in Table 3. As stated, the request, receiving, validation and saving of a segment consumes 5.18 s. However, requesting and receiving one firmware segment via the NB-IoT network consumes only 1.34 s. The verification and saving it into flash memory consumes 6.98 μs and 3.12 s, respectively. The remaining time (0.71 s) is consumed by the MCU to perform all the mentioned activities.

**Figure 13:**
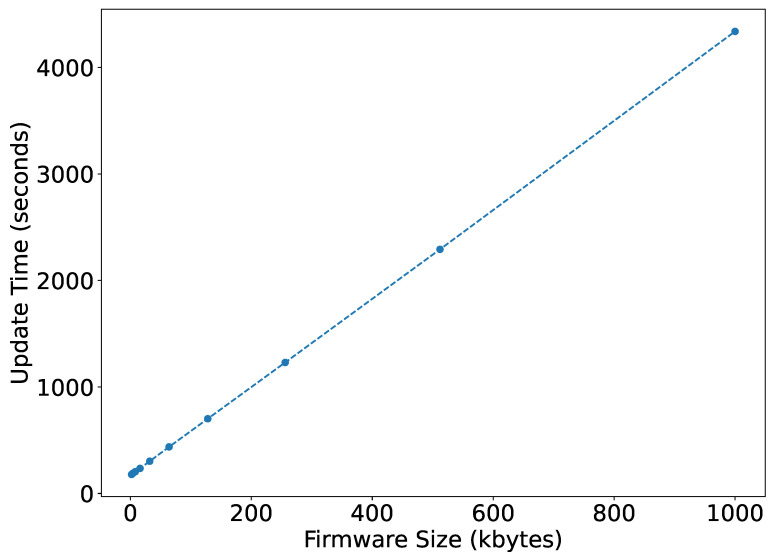
Firmware update time.

Figure 13 shows the overall firmware update time for various firmware sizes. It can be observed to be linear from 178.44 s to 4337.98 s by increasing the firmware size from 2 kB to 1000 kB, whereas for a LoRa network, these update timings vary exponentially. According to the simulations [36,37], using a class C LoRa device at DR5, the update time can vary from 180 seconds to 9 hours by increasing the firmware size from 5 to 200 kB. It means for smaller firmware, both the technology takes similar time. However, with the increase in firmware size, NB-IoT takes much less time than LoRa.

## 5. Discussion

The experiments performed in this work target the firmware update requirements such as version control, integrity, error-free transmission, operability check and reduced user interaction. However, at this moment it lacks authenticity which can be easily implemented by using digital signatures and confidentiality, which can be introduced by encrypting the transmitted update segments. The encryption can triple the latency of each segment [13] and so would affect the battery life.

We use the Orange network in Belgium and Sara N210 NB-IoT module. The results might vary for other operators, as different operators have different algorithms for channel scheduling and link quality adaptation, and so experiences different timings for network acquisition or data transfer. Moreover, different NB-IoT modules, such as Nordic nRF9160 [38] and Quectel BC95-G [39], have slightly different amount of power consumption for different radio states. Therefore, this can affect the presented results about battery life but slightly. When the device is placed such that it receives poor signal strength, the network adapts and assigns lower Modulation Coding Scheme (MCS) values to the UE which results in an increase in latency. The latency of each segment can increase to more than 10 s when the device is placed where the RSSI value is around −110 dBm [40]. Moreover, devices with poor signal strength experience more packet loss and re-transmissions can be observed. However, our experiments tried to avoid packet re-transmissions by placing the device at a location with high signal strength.

## 6. Conclusions

In this paper, we present the framework and implementation for firmware updates on energy-constrained NB-IoT devices. Our design is modular, portable, efficient and can support many features such as differential updates. The implementation is optimized for segment retransmission only when any segment of the firmware update is lost or corrupted in the transmission process (rather than retransmission of the complete firmware). Moreover, we measure the power consumed by the NB-IoT device to perform the firmware update. It can be observed that IoT devices powered by batteries with a capacity of 1500 mAh or more can be updated at a cost of less than 0.75% of their total energy.

## Figures and Tables

**Table 1:** Mailbox sensor application status field.

Status	Signification
OK	The application firmware is updated and valid. The CPU should now jump to the sensor application.
Firmware update request	A firmware update notification is received from the server and the bootloader should perform the firmware update process.
Firmware downloaded	New firmware is downloaded by the bootloader from the server and not yet flashed in the internal flash memory. The bootloader should copy the downloaded firmware into the internal flash memory.
Error	The firmware is corrupted for some reason. The bootloader should again perform a firmware update to recover the corrupted sensor application firmware.

**Table 2:** NB-IoT Messages Format.

Entities	Message Type	Message Format
Application to server	Sensor identity	Sensor#<IMEI>#<IP>#<Software Version>#<Timestamp>
	Measurement	Measurement#<IMEI>#<sensors values>#<Timestamp>
Server to application	Firmware update notification	FirmwareUpdate#<CRC>#<Length>#<Packets number>#<Token>
Bootloader to server	Request Firmware update	RequestFirmwareUpdate#<Token>
	Get Firmware segment	GetSegment#<Index>
	Notify Firmware update status	FirmwareUpdateStatus#<Status>
Server to bootloader	Confirm/reject Firmware update request	FirmwareUpdateConfirm or FirmwareUpdateReject#<Reason>
	Send Firmware segment	<Segment message Id><Length><Index><Data><CRC>

**Table 3:** Power consumption of device at $V_{op}$ = 5 V.

State	Average	Time	Power Con.
	Current Con.	(s)	(mW)
	(mA)		
Initialize, send sensor data and enable FW update	43.488	31.01	217.44
Initialize and Request FW update	38.193	34.02	190.96
Request, receive and save one FW segment	9.044	5.18	44.85
Complete FW CRC and Flash activities	3.134	29.01	15.67
Jump to application (Idle state)	1.924	69.74	9.62
Tx FW update notification	10.311	4.30	51.55

## Data Availability

Not applicable.
